# Contributors to Adherence to Exercise Therapy in Non-Specific Chronic Low Back Pain: A Systematic Review of Qualitative and Quantitative Research

**DOI:** 10.3390/jcm14176251

**Published:** 2025-09-04

**Authors:** Iris Meuwissen, Rob Vanderstraeten, Nathalie Anne Roussel, Mira Meeus, Julie Sylvie Van Eetvelde, Timo Meus, Annick A. A. Timmermans, Jonas Verbrugghe

**Affiliations:** 1 Research Group MOVANT, Department of Rehabilitation Sciences and Physiotherapy (REVAKI), University of Antwerp, 2610 Wilrijk, Belgium; rob.vanderstraeten@uantwerpen.be (R.V.); nathalie.roussel@uantwerpen.be (N.A.R.); mira.meeus@uantwerpen.be (M.M.); julie.vaneetvelde@uantwerpen.be (J.S.V.E.); timo.meus@uhasselt.be (T.M.); jonas.verbrugghe@uhasselt.be (J.V.); 2 Rehabilitation Research Centre (REVAL), Faculty of Rehabilitation Sciences, Hasselt University, 3590 Diepenbeek, Belgium; annick.timmermans@uhasselt.be

**Keywords:** chronic low back pain, adherence, exercise therapy, biopsychosocial, home-exercise program, healthcare professionals, psychosocial factors

## Abstract

**Background/Objectives**: Chronic low back pain is the leading global cause of disability, with a growing prevalence and socioeconomic burden. Despite strong evidence supporting exercise therapy (ET) as a primary treatment, adherence rates remain low, compromising outcomes and increasing healthcare costs. Research on contributing factors to adherence to ET in non-specific chronic low back pain (nsCLBP) is limited. This systematic review aimed to reconceptualise contributors to adherence, using a conceptual framework to explore their interrelations and complexity by integrating quantitative and qualitative research. **Methods**: PubMed, Web of Science and Scopus were searched, followed by a two-phase screening process. Risk of Bias (RoB), certainty assessment and level of evidence were assessed independently. **Results**: Eight qualitative and eleven quantitative studies were included, the latter divided into nine RCTs and two cohort studies. Overall, eight included studies showed low RoB, seven showed some concerns, and four presented high RoB. Synthesis identified internal, external, and intervention-related contributing factors. These factors were presented in a conceptual framework figure, highlighting that adherence should not be viewed as a binary concept but rather as a dynamic behaviour shaped by interrelated factors. Moderate-certainty evidence supports the impact of psychosocial factors, healthcare professional (HCP) characteristics, environmental and time-related factors, program design, progression, home-exercise program (HEP), modalities, and follow-up. Low-to-moderate-certainty evidence suggests beliefs, patient-related characteristics, and treatment setting also impact adherence. Low-certainty evidence indicates that feedback, symptoms and impairments, and confidence possibly impact adherence. **Conclusions**: This systematic review highlights the complex, context-dependent interplay of factors impacting adherence to ET in individuals with nsCLBP. Overall, these findings underscore the need for personalised, context-sensitive interventions that address the broad spectrum of factors, while future research should focus on validated adherence assessment tools.

## 1. Introduction

Chronic low back pain (CLBP), identified by the World Health Organization (WHO) as the leading cause of global disability, imposes a substantial socioeconomic [1] and psychological [2] burden. In the latest edition of the International Classification of Diseases (ICD-11), the WHO, in collaboration with the International Association for the Study of Pain (IASP), classifies non-specific CLBP (nsCLBP) as chronic (duration > 3 months) primary pain, characterised by emotional distress and/or functional disability, not better accounted for by another (secondary) pain condition [3]. Its prevalence is projected to rise from 619 million patients in 2020 to 843 million by 2050, influenced by demographic shifts, such as an ageing population and overall population growth [4]. Consequently, CLBP is one of the most significant cost drivers in healthcare systems worldwide [5].

Clinical guidelines [6,7,8,9] consistently recommend education and exercise therapy (ET) as primary treatment modalities in nsCLBP management. Long-term outcomes appear most favourable when these interventions are delivered within a multidisciplinary program [10]. However, the effectiveness of ET is strongly dependent on patient adherence [11], defined as the extent to which patients follow prescribed exercise programs [10]. Despite robust research supporting treatment efficacy of ET, adherence rates remain low. Current research states that up to 70% of nsCLBP patients lack adherence to prescribed home exercises [12]. Contributing factors include limited health locus of control, lower motivation, insufficient follow-up, lack of supervision, and lower baseline levels of pain and disability [13]. This poor adherence contributes to prolonged disability and drives up healthcare costs [14]. Providing supervised ET, education, goal setting, and follow-up are hypothesised to improve adherence levels in persons with chronic musculoskeletal pain [15].

Recently, a systematic review of qualitative studies [12] identified multiple contributing factors influencing adherence to ET in nsCLBP, concluding that these should not be considered in strictly dichotomous terms. Instead, the authors argued for a reconceptualization of adherence-related factors, highlighting their complex interrelations and considering these factors along a continuum, rather than categorising them solely as dichotomous variables (i.e., hampering or favouring factor) [12]. Unlike theoretical frameworks, conceptual frameworks are better suited to explore relationships in a more flexible and adaptable way, as they allow for a more nuanced representation of dynamic relationships among factors [16]. Moreover, another systematic review demonstrated that many qualitative studies relied on questionnaires not grounded in theoretical frameworks, underscoring the need for alternative methods to synthesise qualitative findings effectively [17].

Existing evidence on adherence-related contributors remains inconsistent, often focusing primarily on biomedical aspects such as postural control and pain intensity [18], or restricted to adherence within home-exercise programs (HEPs) [19]. A recent Delphi study achieved expert consensus on factors to adherence in patients with nsCLBP, i.e., biopsychosocial aspects, competencies of the healthcare professional (HCP), and patient–therapists relationships [20]. However, this study considered only HCP perspectives and exclusively addressed favouring factors.

Mixed-method systematic reviews, integrating both qualitative and quantitative evidence, are particularly recommended for research on multidisciplinary topics, or aim to provide an explanation for possible heterogeneity within trials [21]. Accordingly, this review sought to reconceptualise contributors to adherence, and assess their complexity by implementing a conceptual framework, adapted from the International Classification of Functioning, Disability and Health (ICF) framework, to present a biopsychosocial overview [22], integrating both patients’ and HCP’s perspectives, and combining both quantitative and qualitative research.

## 2. Methods

This systematic review follows the Preferred Reporting Items of Systematic reviews and Meta-analyses guidelines (PRISMA) [23].

### 2.1. Eligibility Criteria

By implementing the PECO (patient, exposure, comparison, outcome) approach, the aim was to conduct an extensive literature search with objective in- and exclusion criteria, reducing the risk of interrater interpretation. All eligibility criteria are shown in Table 1. Only studies written in English and Dutch were included, while case reports, feasibility studies, retrospective studies, and secondary study designs (e.g., systematic review, meta-analyses) were excluded.

### 2.2. Information Sources

Two different databases were used to identify relevant studies: PubMed and Web of Science (WOS). The final search was performed on 9 January 2024. On 4 August 2025, an update of both included databases was performed, and a third database, Scopus, was added to the screening process.

### 2.3. Search Strategy

The combination of free text words and MeSH terms (the latter solely for PubMed search), displayed in Table 2, resulted in comprehensive search strategies for both databases, shown in Appendix A.

### 2.4. Selection Process

The studies were screened by two independent researchers (IM, RV) based on eligibility criteria in two phases using Rayyan Intelligent Systematic Review software (web version) [24]. Phase one of the screening process comprised a title and abstract screening, followed by a full text screening of the remaining studies. Interrater agreements were determined based on percentages and on Cohen’s Kappa calculations (κ), which account for agreement occurring by chance. Discrepancies between reviewers were resolved through discussion.

### 2.5. Data Collection Process

A data extraction table was developed by two authors (IM, RV), thereby providing a standardised overview of the necessary data to extract. Data from the included full-text studies was collected by one researcher (IM) and reviewed by two other researchers (JVE and TM). In case of uncertainties or discrepancies, a fourth author was consulted (JV).

#### Data Items

The following data was extracted from all included studies: author, year of publication, study design, sample (mean ± SD age (years)), eligibility criteria, measurements of adherence, intervention characteristics, duration, follow-up, and contributing factors to adherence. Intervention characteristics were described to compare the different outcomes (contributors) throughout different modalities of ET.

### 2.6. Study Risk of Bias Assessment

Risk of Bias was assessed by two independent researchers (JVE and TM) with the following Risk of Bias (RoB) assessment tools: the Cochrane Risk of Bias Tool for Randomised Controlled Trials (ROB2) [25], and the Critical Appraisal Skills Program (CASP) [26] for qualitative and cohort studies. The full overview of both RoB tools is shown in Appendix C.

For the ROB2, in case of one item being scored either with ‘some concerns’ or ‘high’, their overall score was also scored ‘some concerns’ or ‘high’, respectively. The CASP for cohort studies does not include a scoring model for the overall quality of the study. In line with CASP guidance, it is recognised that the checklist is intended to support structured discussion rather than produce a quantitative score. However, for the purpose of synthesising and comparing risk of bias across a larger number of included studies, a commonly used adaptation in the literature was applied, in which each “Yes” response was scored as 1 point, and “No” or “Can’t tell” as 0. Total scores could therefore range from 0 to 10.

Based on the scientific literature, it was decided that a score of 9–10/10 indicates high quality, a score between 6 and 8 indicates moderate quality, and a score < 5/10 indicates poor quality [25,27,28]. Additionally, if the first two questions are not answered with ‘yes’, the overall score can only be of moderate or high risk [26,29]. Finally, the CASP for qualitative studies does not include a scoring model either. Therefore, the scientific literature and the CASP guidelines were consulted, suggesting the following scoring: >6/10 indicates high quality, a score between 4 and 6 indicates moderate quality, and a score < 4/10 indicates low quality [30,31]. Additionally, if the first two questions are not answered with ‘yes’, the overall score can only be of moderate or high risk [29].

### 2.7. Synthesis Methods

After primary data collection in the evidence table, as described in Section 2.5, outcomes were synthesised per domain, by creating a conceptual framework, adapted from the International Classification of Functioning, Disability and Health (ICF) framework, to present a biopsychosocial overview [22]. After reviewing the (para)medical literature focusing on hampering and favouring factors in regard to the implementation of interventions [32,33,34,35], IM, RV, NR and JV reached a consensus regarding the domain categories. By combining previous research [32,33,34,35] and thorough discussions, the constructs were organised into three categories: internal, external, and intervention-related factors.

Internal factors refer to personal attributes such as beliefs, psychological states, symptoms, and confidence.

External factors include environmental influences, healthcare professional characteristics, and social or contextual elements.

Intervention-related factors encompass aspects of the exercise program itself, such as design, progression, setting, and follow-up.

### 2.8. Certainty Assessment

To evaluate the certainty of the body of evidence of the included quantitative studies, the GRADE (Grades of Recommendation, Assessment, Development and Evaluation) classification method was employed [36,37]. Objective criteria were used to assign a level of evidence based on the following GRADE domains: study design, study quality, RoB, consistency, directness, and other modifying factors, including precision and strength of effect estimates. For the included qualitative studies, the GRADE-CERQual (Confidence in the Evidence from Reviews of Qualitative research) approach was used [38]. The components of this approach consist of methodological limitations, coherence, adequacy of data, and relevance. One researcher (IM) assessed the GRADE level of evidence for each outcome.

## 3. Results

### 3.1. Study Selection

**Figure 1:**
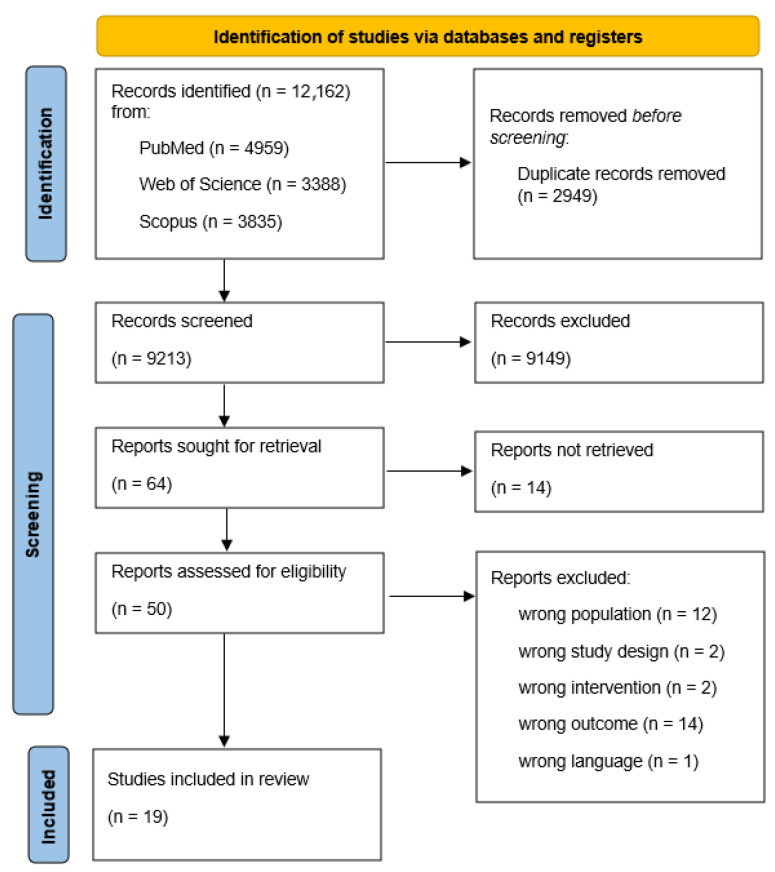
PRISMA flowchart screening process.

Figure 1 presents the PRISMA flow diagram of the search and screening process. The initial search on PubMed and Web of Science on 9 January 2024 yielded 7472 records, with 1643 duplicates removed before screening. The remaining 5829 records were screened by title and abstract, excluding 5771 studies with an interrater agreement of 93.4%. One full-text article could not be retrieved despite requests. Full-text screening of the remaining 44 studies resulted in 16 included primary studies, with an interrater agreement of 90.9%.

An updated search on 4 August 2025 added 855 new studies, and Scopus was included as a third database, contributing 3835 additional records. After combining with the updated previous screening, a total of 4690 records were identified, with 1306 duplicates removed. The remaining 3384 studies were screened by title and abstract. Full-text screening of six studies led to the inclusion of three additional studies. All screening numbers are shown in Figure 1. Across both full text screening rounds, interrater agreement was 90%, with a Cohen’s Kappa of κ = 0.55, indicating moderate agreement. Discrepancies were resolved through discussion between IM and RV.

### 3.2. Study Characteristics

For all included studies, the study characteristics (i.e., author, country, year of publication, sample number, gender, age, intervention characteristics, duration of the follow-up, and contributing factors to adherence) are presented in Table A1, Appendix B.

#### 3.2.1. Population

Participant characteristics of all included studies are shown in Table A1. Nineteen primary studies (n = 1534, mean age = 41.7 years old) were included, of which eight are qualitative [39,40,41,42,43,44,45,46], and eleven are quantitative: nine RCT studies, two cohort ones [47,48,49,50,51,52,53,54,55,56]. Patients were recruited from primary care physicians [39,47,50,51,53], tertiary care hospitals [42,49,51,55,56], outpatient physiotherapy clinics [49,50], university hospital/campus [40,41], inpatient rehabilitation centres [41,48,54], chiropractic offices [45], and through the general public [43,44,46,52,53]. Pain durations and pain intensities of included participants are presented in Table A1 None of the studies provided specific information regarding the ethnicity of the participants.

#### 3.2.2. Intervention Characteristics

In Table A1, Appendix B, a comprehensive overview of all intervention characteristics is shown. Quantitative studies showed an average interventional/observational and follow-up duration of 15.12 weeks, with an average frequency of 2.5× sessions per week; this information was not reported in qualitative studies. Two studies included an ET program [51,52], and three studies compared an exercise program to an additional educational program of patients [47,53,54]. One study evaluated the effect of educated therapists in two exercise groups [50], two studies evaluated the effect of telerehabilitation [55,56], and a single study discussed the added benefit of a motivational program [49], behavioural techniques [57], or a focus on goal setting [48]. Additionally, six studies comprised a home-exercise program [47,49,51,53,54,55,56,57].

#### 3.2.3. Outcome Assessments Linked as Contributors to Adherence

Outcomes assessed by quantitative studies are presented in Table 3. To assess pain and disability, all quantitative studies used validated questionnaires, i.e., four studies used the Roland–Morris Disability Questionnaire (RMDQ) [47,50,51,56], three studies used the (modified) Oswestry Disability Index ((m)ODI) [53,55,57], five used the Numeric Pain Rating Scale (NPRS) [47,49,50,53,56], three used the (back/leg) Visual Analog Scale (VAS) [54,55,57], one used the Patient-Specific Functional Scale (PSFS) [50] and the Low Back Rating Scale (LBRS), and one used the Low Back Outcome Scale Questionnaire (LBOSQ) [49].

Psychosocial outcomes were assessed by questionnaires as well, i.e., one study used the Beck Depression Inventory (BDI) [57], one used the Short-Form 36 (SF36) [55], two used the Tampa Scale of Kinesiophobia (TSK) [54,55], three studies used the Pain Catastrophising Scale (PCS) [51,54,57], four used the Fear Avoidance Beliefs Questionnaire (FABQ) [50,51,53,54], two used the Sports Injury Rehabilitation Beliefs Scale (SIRBS) [48,52], one used the Hospital Anxiety and Depression Scale (HADS) [54], one used the Exercise Self-Efficacy Questionnaire (ESEQ) [51], one used the Health Locus of Control Questionnaire (HLC) [51], one used the Modified Somatic Perception Questionnaire (MSPQ) [51], one used the Treatment Self-Regulation Questionnaire (TSRQ) [50], one used the Behavioural Regulation in Exercise Questionnaire (BREQ-2) [48], one used the European Quality of Life Questionnaire (EurQoL) [50], one used the Depression Anxiety Stress Scale (DASS21) [56], one used the Short Form Health Survey (SF12) [56], and one study used the Patient Satisfaction Questionnaire (PSQ) [57].

Finally, to assess physical outcomes, the Biering–Sørensen test [48], fingertip-to-floor distance [49], Time-up and Go (TUG) [55], Movement System Impaired-Based classification model (MSI) [53], and the International Physical Activity Questionnaire (IPAC) [50] were used.

#### 3.2.4. Outcome Assessments Evaluating Adherence

Additionally, four studies measured adherence by the Sports Injury Rehabilitation Adherence Scale (SIRAS) [48,50,51,52], four used a self-reported questionnaire for adherence rates [47,50,51,53], two used the Exercise Adherence Rating Scale (EARS) [55,56], and two counted the attendance ratio [50,52]. These adherence rates were used in corresponding studies to analyse correlations with assessed contributing factors.

### 3.3. Risk of Bias Assessment

The results for the ROB2, the CASP for cohort studies, and the CASP for qualitative studies are presented in Table 4, Table 5 and Table 6, respectively, with an interrater agreement of 82.8%. Risk of bias was represented using color coding, with low risk, some concerns, and high risk indicated by green, orange, and red, respectively.

Overall, five RCT studies [47,51,53,55,56] displayed moderate RoB concerns, while the other four RCT studies [48,49,50,52] had a high RoB. Both cohort studies included [54,57] received the score ‘low risk of bias’. Comments regarding the decisions are presented below the table. Finally, regarding qualitative studies, two [40,44] scored a moderate risk of bias, while the other six studies [39,41,42,43,45,46] scored a low risk of bias.

Additionally, researchers experienced differences in interpretation, specifically:

1. ROB2: disagreements over missing data and blinding procedures, which are subjective and can be interpreted in different ways.
2. CASP cohort studies: one researcher was more critical about how exposure was measured, leading to different interpretations.
3. CASP qualitative studies: disagreement on two studies [41,43] due to inadequate information for scoring the criteria for one of the reviewers.

### 3.4. Results of Synthesis: Favouring and Hampering Factors

**Figure 2:**
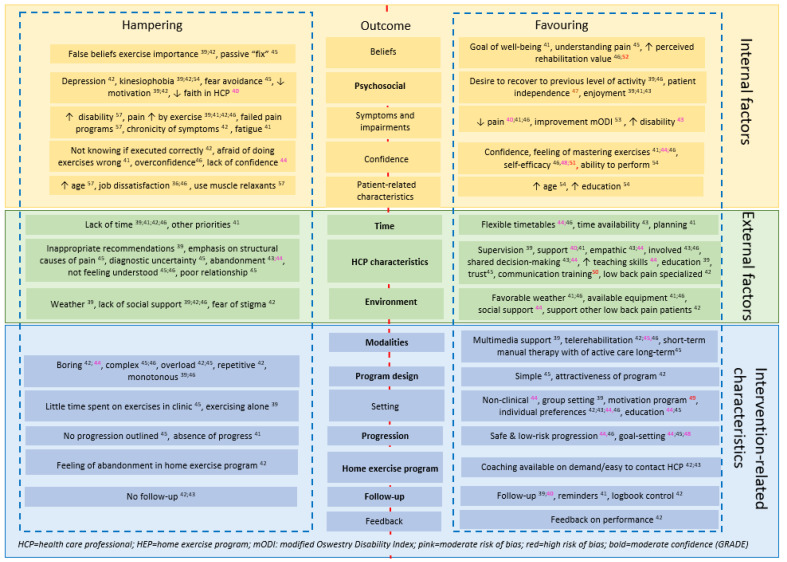
Conceptual framework of quantitative and qualitative studies.

Figure 2 presents a comprehensive overview of the contributors to adherence to ET in patients with nsCLBP, encompassing both hampering and favouring factors. These constructs are organised into three primary categories: internal factors, external factors, and intervention-related characteristics, as described in Section 2.8.

In the subsequent synthesis, the findings are reported according to the quality of the included studies. Quantitative and qualitative findings are discussed based on the GRADE and GRADE-CERQual, respectively, as outlined in Section 3.5.

#### 3.4.1. Internal Factors Impacting Adherence

##### Quantitative Evidence

Moderate-confidence evidence (n = 1) reported a psychosocial factor, namely, patient independence [47], as positively impacting adherence (*p* = 0.001). Low-confidence evidence described increased perceived rehabilitation value [52] as a favouring factor to adherence (*p* < 0.05). Low-confidence evidence (n = 1) described confidence, i.e., ability to perform exercises, as favouring adherence [54] (*p* < 0.05). Additional low-confidence evidence (n = 1) shows symptoms and impairments, i.e., greater disability has been shown to both hamper [57] (*p* < 0.000) and favour [53] (*p* = 0.05) adherence. Finally, low-confidence evidence (n = 2) described patient-related characteristics as, respectively, favouring (i.e., increased age (*p* = 0.018) and education (*p* < 0.001) [54]), and hampering adherence (i.e., increased age (*p* = 0.03) and the use of muscle relaxants (*p* = 0.005) [57]).

##### Qualitative Evidence

Moderate-confidence evidence (n = 3) identified several beliefs to favour adherence, i.e., goal of well-being [41], perceived rehabilitation value [46], and understanding pain [45]. Furthermore, moderate-confidence evidence (n = 4) highlights psychosocial factors to positively impact adherence, i.e., enjoyment [39,41,43], and the desire to recover to the previous level of activity [39,46]. Finally, low-confidence evidence (n = 4) describes symptoms and impairments, i.e., decreased pain [40,41,46], and confidence, i.e., feeling of mastering exercises [41,44,46] and self-efficacy [46], to favour adherence.

In regard to internal factors hampering adherence, moderate-confidence evidence (n = 3) identified beliefs, i.e., false beliefs on exercise importance [39,42], and focus on a passive fix [45]. Additionally, moderate-confidence evidence (n = 2) described patient-related characteristics, i.e., job dissatisfaction [39,46], to hamper adherence. Moderate-confidence evidence (n = 4) highlighted several psychosocial factors to hamper adherence as well, i.e., depression [42], kinesiophobia [39,42], fear-avoidance [45], decreased motivation [39,42], and decreased faith in HCP [40]. Low-confidence evidence (n = 1) described confidence, i.e., overconfidence, as hampering adherence [46]. Finally, low-confidence evidence (n = 4) identified symptoms and impairments, i.e., aggravated pain by exercise [39,41,42,46], chronicity of symptoms [42], and fatigue [41].

#### 3.4.2. External Factors Impacting Adherence

##### Quantitative Evidence

Moderate-confidence evidence (n = 1) described a significant, positive correlation (*p* = 0.01) to favour adherence to ET when HCP’s received communication training [50]. No external factors hampering adherence were described in quantitative studies.

##### Qualitative Evidence

Moderate-confidence evidence (n = 6) described several other HCP characteristics as external factors favouring adherence, i.e., supervision [39], support [40,41], and empathic skills [43,44], applying shared-decision making [43,44], teaching skills [44], being involved [43,46], education [39], building trust [45], and specialised LBP [42]. Furthermore, moderate-confidence evidence (n = 4) identified favouring time factors, i.e., flexible timetables [44,46], time availability [43], and planning [41]. Finally, moderate-confidence evidence (n = 4) described favouring environmental factors, i.e., favourable weather [41,46], available equipment [41], social support [44], and support of other CLBP patients [42].

In regard to external factors hampering adherence, moderate-confidence evidence (n = 5) identified HCP characteristics, i.e., HCP’s emphasis on structural causes of pain [45], inappropriate recommendations [39], diagnostic uncertainty [45], abandonment [43,44], not feeling understood [45,46], and a poor patient–therapist relationship [45]. Additionally, moderate-confidence evidence (n = 4) highlighted time factors, i.e., lack of time [39,41,42,46] and other priorities [41]. Finally, moderate-confidence evidence (n = 3) highlighted the hampering effects of environment, i.e., weather [39], fear of stigma [42], and lack of social support [39,42,46].

#### 3.4.3. Intervention-Related Factors Impacting Adherence

##### Quantitative Evidence

Moderate-confidence evidence (n = 1) described a significant, positive correlation (*p* < 0.05) to favour adherence to ET regarding progression, when participants received clear goal setting [48]. Moderate-confidence evidence (n = 1) described modalities (i.e., the implementation of telerehabilitation), another significant, positive relation (*p* < 0.05) to favour adherence [55]. Low-confidence evidence (n = 1) mentioned setting, i.e., the implementation of a motivational program, to favour adherence (*p* < 0.05) [49]. No hampering factors were described by quantitative studies.

##### Qualitative Evidence

Moderate-confidence evidence (n = 6) described modalities, i.e., multimedia support [39], telerehabilitation [42,46], or short-term manual therapy [45], to favour exercise adherence. Moderate-confidence evidence (n = 2) mentioned program design to positively impact adherence (i.e., simple [45] and the attractiveness of the program [42]). Moderate-confidence evidence (n = 6) highlighted the importance of the setting, i.e., non-clinical [44], group setting [39], implementing individual preferences [42,43,44,46] and education [44,45]. Additionally, moderate-confidence evidence (n = 3) described progression, i.e., safe and low-risk progression [44,46], and goal setting [44,45]. Moderate-confidence evidence (n = 2) presented home-exercise characteristics, i.e., coaching available on demand (with an easy to contact HCP) [42,43] to positively impact adherence. Moderate-confidence evidence (n = 4) described follow-up, i.e., reminders [41], logbook control [42], and follow-up [39,40] to favour adherence. Finally, low-confidence evidence (n = 1) highlighted the importance of feedback on performance [42].

Furthermore, in regard to intervention-related factors hampering adherence, moderate-confidence evidence (n = 5) identified that a boring or complex program design resulted in participants being less adherent [39,42,44,45,46], and moderate-confidence evidence (n = 2) indicated a lack of follow-up [42,43] to hamper adherence. Additionally, moderate-confidence evidence (n = 2) described the hampering effect of settings, i.e., little time spent on exercises in clinic [45] and exercising alone [39]. Moderate-confidence evidence (n = 1) identified a lack of outlined progression [45], as well as absence of progress [41], and, finally, low-confidence evidence (n = 1) highlighted the hampering effect of feeling of abandonment in the home-exercise program [42].

### 3.5. Certainty of Evidence

All outcomes have been assessed for their level of evidence, and, as previously described, these results are separated into quantitative (GRADE) and qualitative research (GRADE-CERQual), presented in, respectively, Table A2 and Table A3, Appendix C.

#### 3.5.1. Quantitative Studies (GRADE)

In internal factors, four outcomes were graded with a low-confidence level (i.e., beliefs [52,57], symptoms and impairments [47,53,57], confidence [48,51,54], and patient-related characteristics [47,54,57]), while psychosocial factors received a moderate-confidence level [47,54]. Furthermore, the sole outcome of external factors, HCP characteristics [50], received a moderate-confidence level. Finally, in intervention-related characteristics, one outcome (setting [49]) received a low-confidence level, while modalities [55] was graded with a moderate-confidence level, and progression [48] received a moderate-confidence level.

#### 3.5.2. Qualitative Studies (GRADE-CERQual)

Three internal factors were scored as moderate-confidence level as well, i.e., beliefs [39,41,42,45,46], psychosocial [39,40,41,42,43,46], and patient-related characteristics [39,41,46], while two other internal factors were scored as low-confidence level, i.e., symptoms and impairments [39,40,41,42,46] and confidence [41,42,44,46]. Furthermore, all three external factors were graded with a moderate-confidence level, i.e., HCP characteristics [39,40,41,42,43,44,45,46], environment [39,41,42,43,46], and time [39,41,42,43,44,46]. Finally, six intervention-related characteristics were scored as moderate-confidence level, i.e., modalities [39,42,45,46], program design [39,42,44,45,46], setting [39,42,43,44,45,46], progression [44,45,46], HEP [42,43], follow-up [39,40,41,42,43], while one was scored as low-confidence level, i.e., feedback [42].

## 4. Discussion

Treatment adherence is widely recognised as a key determinant of therapeutic success. However, the underlying contributors are complex and multifactorial. Adherence is often reduced to a simplistic binary classification which, while useful in some contexts, may oversimplify the complex, nuanced, and dynamic nature of adherence behaviour [12]. To address this gap, the present systematic review explored factors impacting adherence to ET among individuals with nsCLBP, presented on a continuum.

Moderate-certainty evidence was found for multiple factors impacting adherence. These include psychosocial factors [39,40,41,42,43,45,46,47,54], HCP characteristics [39,40,41,42,43,45,46,50], environmental conditions [39,41,42,44,46], time-related considerations [39,41,42,43,44,46]; progression [44,45,46,48], program design [39,42,44,45,46], modalities [39,42,45,46,55], follow-up strategies [39,40,41,42,43] and HEP [42,43].

Low-to-moderate-confidence evidence suggests that beliefs [39,41,42,45,46,52,57], symptoms and impairments [39,40,41,42,46,53,57], patient-related characteristics [39,41,42,45,46,52,57], and setting [39,42,43,44,45,46,49] may also impact adherence to ET. While the certainty of evidence from quantitative studies was rated as low according to the GRADE framework, qualitative evidence was judged to have moderate certainty, highlighting potential inconsistency across evidence types.

Finally, there is low-certainty evidence that feedback [42] favours adherence and confidence [41,44,46] possibly impacts adherence in both directions.

### 4.1. Interpretation of Main Findings and Implications for Clinical Practice

This review demonstrated that adherence is a complex outcome impacted by a wide range of contributors. Building on the conceptual framework presented in Figure 2, these factors, relating to internal, external, and intervention-specific characteristics, can be organised into seven overarching constructs that are highly relevant to clinical practice, merging both quantitative and qualitative outcomes. These constructs include: (1) psychological contributors and the added value of Pain Science Education (PSE), (2) skills of the HCP, (3) environmental factors, (4) implementing goal setting, (5) achieving shared-decision making, (6) creating a context-specific exercise program, and (7) integrating patient-specific factors into the program. Each of these will be discussed in the following sections, ordered by the strength of the supporting evidence.

#### 4.1.1. Psychological Contributors and the Added Value of Pain Science Education (PSE)

As expected, multiple studies confirmed that psychological barriers (i.e., depression, kinesiophobia, fear-avoidance beliefs, reduced motivation, a perceived lack of progress and increased pain during the exercise) were associated with decreased adherence. These findings underscore the importance of adequate pain (science) education (PSE), aimed at provision of reassurance prior to ET, to induce behavioural change [58,59]. PSE, as indicated by several guidelines, including those of the WHO, should be implemented in treatment plans. Ideally, PSE should be provided in a personalised manner with enjoyable exercises, similar to the individual tailoring of ET programs [58,59,60]. PSE appears to be invaluable, since it focuses not solely on explaining the neurophysiology of pain, but also on both biological and psychological factors, in order to reconceptualise patients’ beliefs on, e.g., pain–tissue damage relationships, and the physical activity short term-long term paradox [61,62]. Patients need to understand that physical activity is not inherently harmful and that the experience of pain is not necessarily a contraindication for continuing exercise [58,59]. In contrast, ET in patients with chronic musculoskeletal pain is increasingly provided in a time-contingent manner, rather than a pain-contingent manner, indicating that pain intensity is not the main determinant in executing the exercises [63].

However, before patients can adhere to this time-contingent approach, it is essential that they understand the underlying mechanisms, for which PSE is indispensable. Explaining exercise-induced hypoalgesia, and the interrelation with beliefs such as fear-avoidance and catastrophising, enhances the patients’ understanding of the complexity of nsCLBP [64,65]. To maximise adherence, strategies should not only integrate PSE, but also incorporate behaviour change techniques such as motivational interviewing and goal setting to address fear, low motivation, and maladaptive beliefs in clinical practice [66,67].

Nonetheless, physiotherapists frequently report time constraints as a key barrier to delivering effective patient education [68], and its effectiveness largely depends on empathic, support, teaching- and communication skills of the HCP, as discussed in Section 4.1.2. These findings emphasise that interventions aimed at improving adherence must address both patient-related psychological barriers and the way education is delivered by the HCP.

#### 4.1.2. Skills of the HCP

While patient-related factors play a critical role in adherence to ET, this review underscores the equally significant role of the HCP. Consistent with findings in other chronic pain populations, factors such as empathic communication, clinical expertise, and a supportive therapeutic relationship emerged as key facilitators of adherence [46,69,70]. The HCP’s level of training, particularly when specialised in managing low back pain, also appears to be a favouring factor, reinforcing the importance of clinical expertise in guiding patients through exercise-based interventions. Furthermore, HCPs who demonstrate strong interpersonal skills and provide individualised, supervised treatment appear better able to foster trust and engagement. Conversely, poor communication, lack of empathy, and diagnostic uncertainty were commonly associated with decreased adherence. These findings align with previous meta-analytic evidence, showing a 19% increased risk of non-adherence when HCP communication was lacking [70]. The importance of the patient–therapist relationship has been highlighted as one of the most critical determinants of adherence [20,46,71], reinforcing the need for relationship-centred care and shared decision-making in exercise-based interventions for CLBP [72]. These results indicate that interventions to improve adherence should not only focus on patient education and behaviour change techniques, but also actively strengthen HCP communication and counselling skills. Structured training in empathic communication, shared decision-making tools, and innovative delivery models (e.g., digital PSE platforms) may help overcome time constraints while supporting high-quality patient–therapist interactions [73,74].

#### 4.1.3. Environmental Factors

Practical and environmental factors were additionally identified to influence adherence. Flexible scheduling, structured planning, and sufficient available time have been associated with improved adherence, whereas time constraints and competing priorities were frequently reported as barriers. Notably, time constraints are even described in previous research as one of the biggest barriers to physical activity in multiple populations, including elderly, athletes, and patients with CLBP [75,76].

Facilitating environmental factors include favourable weather conditions [41,46], access to appropriate equipment [41], and peer support from others with low back pain [42]. Conversely, a lack of social support [39,42,46] and fear of stigmatisation [42] emerged as environmental barriers to sustained engagement in ET.

#### 4.1.4. Implementing Goal Setting

In order to provide exercise progression of the intervention, the implementation of goal setting increases the level of individual tailoring in a treatment plan. This approach aligns closely with the implementation of individual preferences [42,43,44,46]. However, only 8% of treatment plans provide high-tailoring, while 81% provide low-tailoring in chronic musculoskeletal pain [77]. Even though high-tailoring is known to significantly improve disability and pain, this discrepancy reveals a clear gap in clinical implementation [78]. Notably, patients have described “disagreement between team and patient on the treatment content”, as one of the reasons to stop the treatment program, further emphasising the importance of aligning treatment with individual preferences [46,78].

#### 4.1.5. Achieving Shared-Decision Making

The results of this review highlight the importance of involving the patient as an active partner when designing exercise programs. This supports the integration of shared-decision making, a process shown to enhance adherence by aligning treatment strategies with patient preferences and values [79].

Enjoyment of exercise has emerged as a recurring positive contributor to adherence across several studies. This aligns with findings that tailoring exercise programs to individual preferences, including the implementation of telerehabilitation or multimedia, can support adherence [55,56,68,80,81] and increase the attractiveness of the intervention. Other facilitators include the desire to return to a previous level of physical functioning and motivation, as confirmed by earlier research [46,58]. Conversely, exercise programs perceived as boring, repetitive, simple or monotonous have been consistently associated with reduced adherence to exercise [82]. In order to provide an accurate answer to the question of whether or not complex exercises favour or hamper adherence, an overarching factor needs to be implicated. Specifically, we need to take a closer look at the confidence of the patient, which, in this review, only showed low-confidence evidence. Several qualitative studies have mentioned the effect of confidence in exercises, e.g., the feeling of mastering exercises increased adherence [41,46], while not knowing if the exercises are executed correctly [42] and being afraid of doing exercises wrong [41] hampered adherence. These results appear to be closely related to the difficulty level of exercises, where an overly complex program could indeed hamper adherence, but when patients feel confident in their exercises and know how to perform them, a rather more complex exercise program increases adherence [42,46].

Furthermore, according to a recent systematic review, only 39% of patients feel actively involved in choices regarding their healthcare, whereas 37% feel less involved than they would like to be [83]. Nonetheless, almost all patients want to be actively involved in decisions regarding their healthcare [83,84]. This discrepancy may partly stem from insufficient training in shared decision-making among HCPs [85], possibly hindering the exchange of information and the articulation of treatment preferences by both HCPs and patients, thereby impeding the achievement of shared decision-making. This limitation is hypothesised to result due to deficits in communication skills, which were also identified as influential factors in this review [83,84].

#### 4.1.6. Creating a Context-Specific Exercise Program

Most evidence on contributors to adherence focuses on intervention characteristics, including program design, exercise modalities, home-based options, and follow-up appointments. These features can strongly favour or hamper patient adherence and should guide the development of context-specific training programs.

One key element shown to support adherence is the inclusion of on-demand coaching within HEPs [42,43]. Personalised support and the ability to receive guidance when needed appear to enhance motivation and ensure correct exercise execution [86]. However, practical implementation of such individualised coaching is often limited by financial constraints and logistical challenges, i.e., the unavailability of a coach nearby, making widespread use in routine clinical settings challenging [42].

In this context, telerehabilitation emerges as a promising and cost-effective alternative, applicable within HEPs or as an adjunct to ET [81]. It has demonstrated efficacy in improving outcomes in other chronic musculoskeletal conditions [87], and its potential in patients with nsCLBP warrants further study [42].

The studies included in this review initially appeared to show mixed results regarding its effect on adherence. One RCT [56] reported no significant effect on EARS scores but noted lower dropout rates in the intervention group (5% vs. 18%), potentially due to increased enjoyment from the dynamic video support [56]. Furthermore, participants in both groups reported relatively high EARS scores (44–49/64), both on initial assessment and follow-up, indicating a ceiling effect that may have limited the ability to detect between-group differences. This ceiling effect has previously been described in studies validating the EARS, which may support this interpretation [88,89]. In contrast, another RCT [55] did report a significant positive effect on EARS scores, likely facilitated by lower baseline scores (CG 12.5, EC 21.3), allowing for greater improvement potential.

Beyond logistical advantages, telerehabilitation can provide real-time feedback and visual guidance, both of which have been reported as favouring adherence [42,46,56]. Similarly, mobile health interventions, particularly smartphone applications, may enhance engagement, reduce healthcare costs, and overcome geographic barriers [55,56,90]. However, most commercially available apps lack clinical validation and standardisation, which limits their evidence-based application [91].

Finally, reminders and follow-up remain critical components, positively influencing adherence, particularly when combined with coaching. Despite their effectiveness, follow-up is often underutilised, implemented in only 41% of treatment plans [92], and patient engagement during follow-up is frequently poor (e.g., 46% of patients failed to complete required questionnaires) [78]. Given the strong evidence supporting the role of follow-up in sustaining long-term outcomes [92], its inclusion is clinically indispensable. A combination of behavioural prompts, reminders, and accessible guidance may be especially effective in reinforcing [93].

#### 4.1.7. Integrating Patient-Specific Factors into the Program

Although supported by limited evidence, several internal factors have been associated with increased adherence to ET, including self-efficacy, confidence, the feeling of mastering exercise, and the perceived ability to perform the exercises. Higher levels of self-efficacy and self-confidence may promote more autonomous execution of exercises [94]. This autonomy could partly account for the positive relationship observed between adherence and the ability to perform independently [95]. Notably, a substantial proportion of individuals with nsCLBP exhibit poor self-efficacy, with prevalence rates reaching 64%, and up to 75% among females [96]. Exercise interventions can improve pain self-efficacy in adults with nsCLBP [97], which, in turn, promotes higher activity levels and greater work endurance [98].

Demographic factors, such as education and age, also influence adherence. Higher education consistently favours adherence, likely due to greater health literacy and understanding of treatment rationale [99]. However, age effects are mixed: one study identified older age to favour adherence, possibly due to fewer external stressors or greater availability of time in older individuals [54]. In contrast, another study found that older age was associated with reduced adherence, reflected in higher dropout rates [57]. Differences may reflect contextual factors such as variations in healthcare delivery systems (Belgium vs. USA), recruitment strategies (general hospital vs. spine rehabilitation centre), and the 25-year gap between both publications. Comorbidities and degenerative spinal changes further complicate adherence in older adults, highlighting the need for age-specific research [100].

Digital literacy and technology access shape adherence to telerehabilitation, particularly in older adults or populations with limited prior exposure. Barriers include limited digital skills, interface complexity, and unreliable internet [92]. To ensure equitable access and sustained adherence, it is crucial that digital exercise interventions are designed with user-centred principles, provide adequate onboarding and support, and offer alternative formats where needed [92].

To illustrate how adherence-related factors can be conceptualised along a continuum, the implementation of telerehabilitation in patients with nsCLBP is considered as an example. Telerehabilitation is often viewed as either a hampering or favouring factor to adherence. However, its impact is highly context-dependent and varies across individuals based on a spectrum of interrelated factors. For example: Patient A has high digital literacy, reliable internet access, strong self-efficacy, and a supportive home environment. This patient navigates the telerehabilitation platform with ease, engages consistently with the prescribed exercises, and benefits from features such as reminders and real-time feedback. In this context, telerehabilitation acts as a strong favouring factor of adherence [101,102]. Patient B, on the other hand, has limited experience with technology, poor internet connectivity, low motivation, and minimal social support. This patient struggles to use the platform, misses sessions due to technical issues, and feels isolated during the rehabilitation process. For this individual, telerehabilitation becomes a hampering factor to adherence [101,102].

This example demonstrates that telerehabilitation should not be classified as simply “effective” or “ineffective.” Instead, its influence on adherence exists along a continuum shaped by technological, psychological, and environmental factors. Recognising this spectrum allows clinicians to tailor interventions more effectively, ensuring that digital tools are matched to the patients’ capabilities and context.

By organising contributing factors into internal, external, and intervention-related domains, the conceptual framework encourages clinicians to move beyond a binary view of adherence and instead consider it a dynamic, context-sensitive process. This perspective supports more personalised treatment planning by helping clinicians identify and address modifiable contributors to adherence across multiple levels, ranging from patients’ beliefs and psychosocial profiles to environmental constraints and program design features.

### 4.2. Strengths and Limitations and Suggestions for Further Research

A key strength of this review is its mixed-method approach, integrating quantitative and qualitative studies to provide a more comprehensive understanding of adherence by highlighting the added value of qualitative research, particularly its capacity to capture nuanced and underexplored outcome domains often absent in quantitative designs. Notably, this systematic review distinguishes itself by categorising contributors to adherence into internal, external, and intervention-related factors, followed by adopting a biopsychosocial, context-dependent discussion, providing relevant clinical implications. While previous studies often categorised factors dichotomously [17], this review emphasised how the same factor can act as both hampering and favouring depending on context, reflecting the complex nature of adherence, and provides a foundation to recommendations for clinical practice. However, this conceptual framework was not formally validated, which may introduce interpretation bias and should therefore also be considered a limitation.

Furthermore, this review highlights factors that, up until recently, have been underexplored, e.g., factors contributing to the effects of shared-decision making and home-exercise programs. Finally, this review provides preliminary results and shortcomings in both the current literature and guidelines, and, therefore, demonstrates outcomes of clinical relevance. However, even though this review showed methodological strengths, it did show a limitation regarding data extraction, which was performed by one researcher, and thoroughly checked by the others.

One notable limitation in the current literature is the frequent use of dropout rates as a proxy for adherence, a practice that has been widely critiqued. An umbrella review emphasised that adherence should not be reduced to mere program attendance or completion [82]. Instead, it should be defined by the degree to which a patient’s behaviour aligns with the agreed-upon treatment plan [82]. This critique highlights a broader issue: the considerable variability and inconsistency in how adherence is defined and measured across studies. In supervised settings, adherence is often gauged using attendance ratios or rating tools such as the SIRAS, an adherence assessment method rated by HCPs [103] or Likert scales. For HEPs, self-reported diaries are commonly used [93]. However, such methods may primarily capture compliance, defined as passive following of HCP instructions, rather than adherence, which involves an active, motivated engagement by the patient. To better capture this complexity, more comprehensive and validated instruments that also assess underlying psychosocial factors like motivation are needed. One promising development is the Adherence To Exercise for Musculoskeletal Pain Tool (ATTEMPT), recently designed using input from patients, clinicians, and researchers. While initial validation is promising, further studies are needed to assess its construct validity and responsiveness [104].

Furthermore, the limitations of this review include the lack of high-quality evidence, including limited data on long-term adherence, creating a gap in the literature, and thereby providing an opportunity for further researchers. Additionally, although the CASP tool does not provide a scoring system or formal thresholds, an adapted approach was implemented to facilitate comparison across studies. While this scoring facilitates synthesis, the authors acknowledge it may not capture the full nuance of individual study appraisal as intended by CASP.

Lastly, discrepancies between self-reported and objective exercise data in chronic pain populations highlight the urgent need for more behaviourally sensitive, objective adherence measures in future research, especially in unsupervised settings [105]. Future research should prioritise the development and validation of such tools, e.g., ATTEMPT, to enable more reliable and actionable assessments of patient engagement.

## 5. Conclusions

This review provides a comprehensive synthesis of the complex and multifactorial impact on adherence to ET in individuals with nsCLBP. While adherence is often viewed in binary terms, adherent or non-adherent, our findings emphasise the importance of understanding adherence as a dynamic and nuanced behaviour shaped by multiple interacting factors.

Moderate-certainty evidence supports the role of external, e.g., HCP characteristics, and psychosocial factors as contributors to adherence, as well as intervention-related elements, such as modalities. Low-to-moderate-certainty evidence further suggests that several internal factors and treatment setting may impact adherence, though the certainty of evidence from quantitative studies was generally low. Qualitative findings were rated with moderate confidence, highlighting a disparity in evidence strength between research approaches. Additionally, preliminary evidence suggests that feedback and patient confidence may either facilitate or hinder adherence depending on context.

Finally, overarching constructs that provide direct clinical implications have been identified, including psychological contributors and the added value of PSE, skills of the HCP, environmental factors, implementing goal setting, achieving shared-decision making, creating a context-specific exercise program, and integrating patient-specific factors into the program.

Overall, these findings underscore the need for personalised, context-sensitive interventions that address the broad spectrum of factors contributing to adherence. Future research should prioritise the development and validation of tools to objectively assess patient adherence. One promising development is the recently designed ATTEMPT, using input from patients, clinicians, and researchers, which requires further research to determine its construct validity and responsiveness.

## Figures and Tables

**Table 1:** PECO eligibility criteria.

	Population	Exposure	Comparison	Outcome
**Inclusion criteria**	Chronic non-specific low back pain	Contributors to adherence	/	Adherence to exercise therapy
**Exclusion criteria**	(Sub)acute pain, specific low back pain, neck or thoracic (solely) Fibromyalgia or other specific chronic pain/musculoskeletal/neurological conditions Children (i.e., <18 year) Experimentally induced LBP	/	/	Healthcare professionals’ view on adherence Solely adherence rates without description of contributors

**Table 2:** PECO search strategy.

	Population	Exposure	Comparison	Outcome
**Free keywords**	Chronic AND (low OR lower OR spinal OR lumbar OR lumbalgia OR spine OR back) AND Pain	Exercise OR training OR telerehabilitation OR self-management	/	Compliance OR adherence OR compliancy OR contributor* OR barrier* OR facilitator* OR motivation OR knowledge OR attitude* OR belief* OR behavi* OR awareness
**MeSH terms**	Pain OR Back pain OR Low back pain	Exercise therapy OR Exercise OR Exercise movement techniques OR Telerehabilitation OR Self-management	/	Attitude to health OR Health belief model

**Table 3:** Evidence table of quantitative studies.

Reference Author (Country; Year)	Intervention	Outcome Assessment	Results	Contributors (+ = Favouring Adherence; − = Hampering Adherence)
Azevedo (Brazil; 2021) [47]	MSI	Self-reported adherence in diary (daily, during the 8-week treatment)	Group 1: ↑ patient independence in home exercises = ↑ adherence to treatment (*p* = 0.001) Group 2: ↑ RMDQ = ↑ adherence in home exercises (*p* = 0.05)	○ Patient independence (+) ○ More disability (+)
Coppack (UK; 2012) [48]	Goal setting and ET	SIRAS (three times per week, for three weeks; mean value across the nine appointments)	○ Exp > C2 SIRAS ↑ (*p* < 0.025) ○ Exp > C1 and C2 SIRBS ↑ (*p* < 0.05) ○ Both = positive effects of goal setting and pt–therapist relationship on self-efficacy and adherence; indicating indirect relationship between increased self-efficacy and adherence	○ Goal setting (+) ○ Self-efficacy (+)
Dhondt (Belgium; 2020) [54]	Individual multimodal rehabilitation program	Registered treatment attendance (daily, for 24 weeks (36 sessions))	○ ↑ age = ↑ adherence (*p* = 0.018) ○ ↑ VAS low-load activities = ↑ adherence (*p* = 0.030) ○ ↑ TSK = ↑ adherence (*p* < 0.001) ○ ↓ education level = ↓ adherence (*p* < 0.001) ○ No poor-posture-related LBP = ↓ adherence (*p* = 0.028)	○ Higher age (+) ○ Self-reported ability to perform low-load exercises (+) ○ Higher education (+) ○ Kinesiophobia (−)
Feng (China; 2025) [56]	Education, coaching, ET through mobile health (mHealth)	Exercise Adherence Rating Scale (EARS) (baseline, after 4 weeks (during treatment) and after 8 weeks (post treatment)	Exp = CG adherence (*p* = 0.06)	○ No significant contributors
Friedrich (Austria; 1998) [49]	Combined exercise and motivation program	Recorded compliance with prescribed exercise regimen, daily exercise diary (every session, and at 4- and 12-month follow-up)	○ Exp > contr. attendance therapy sessions (*p* = 0.0005) ○ Exp > contr. 12-month compliance to weekly training frequency (*p* = 0.036) ○ ↑ initial level of distress = ↑ total training time at 4- and 12-month follow-up ○ ↑ initial level of internal control = ↑ total training time at 4- and 12-month follow-up ○ Positive exercise attitude = ↑ attendance physical therapy sessions	○ Motivation program (+) ○ Higher level of initial distress (+) ○ Higher level of initial internal control (+) ○ Positive exercise attitude (+)
Lonsdale (Ireland; 2017) [50]	CONNECT	Self-reported home-based adherence (pt), SIRAS, % completed sessions (T0 = baseline, T1 = week 1, T2 = week 4, T3 = week 12, T4 = week 24)	Exp (communication training PT) = ○ ↑ adherence (short-term) (*p* = 0.01) ○ ↑ clinical outcomes ♀ ○ ↑ motivation (*p* = 0.01)	○ Communication training physiotherapists (+)
Mannion (Switzerland; 2009) [51]	Spine segmental stabilisation exercises	MAI (averaged by combining daily self-reported adherence, SIRAS (each therapy session), % attendance)	Self-efficacy = sign correlated with MAI (*p* = 0.045)	○ Self-efficacy (+)
Owen (Australia; 2022) [52]	MCMT and GSC	SIRAS, attendance ratio (measured at baseline (T0) and at 6 months (end of interventional period; T1)	GSC group: perceived rehabilitation value predicted adherence behaviour (*p* = 0.048)	○ Higher perceived rehabilitation value (+)
Özden (Turkey; 2022) [55]	Video exercise–based telerehabilitation software (Fizyoweb)	Exercise Adherence Rating Scale (EARS)(baseline, after 4 weeks (during treatment) and after 8 weeks (post treatment))	Exp > CG EARS (*p* = 0.0001)	○ Telerehabilitation (+)
Rainville (USA; 1997) [57]	ET and behavioural techniques	Number of dropouts (T1 = 12 months)	Dropout group: ○ higher mean ODI score (*p* < 0.000) ○ higher Back-VAS score (*p* = 0.02) ○ higher age (*p* = 0.03) ○ failed pain programs (*p* = 0.05) ○ use muscle relaxants (*p* = 0.005)	○ Higher disability score (−) ○ Higher pain score (−) ○ Higher age (−) ○ Failed pain programs (−) ○ Use muscle relaxants (−)
Van Dillen (USA; 2016) [53]	NC and CS	Self-reported adherence (% of exercises performed) at baseline (T0), after the last treatment visit (T1), and 6 (T2) and 12 months (T3) later	No change in performance training adherence at post-treatment (*p* > 0.05); performance training adherence declined less (79–62%) than exercise adherence (80–40%) in both groups Greater improvement mODI score = ↑ performance training adherence (*p* < 0.05) and vice versa	○ Greater improvement mODI score (+)

♀ = female; ↑ = increased; ↓ = decreased; + = favouring adherence; − = hampering adherence; ARP = Activity-Related Pain questionnaire; BARSE = Barriers Self-Efficacy Scale; BBQ = Back Belief Questionnaire; BDI = Beck Depression Inventory; BIPQ = Brief Illness Perception Questionnaire; CG = Control Group; CNS = Central Nervous System; CONNECT = Communication Style and Exercise Compliance in Physiotherapy; CS = Classification-Specific Treatment; EARS = Exercise Adherence Rating Scale; EBPC = Evidence-Based Physiotherapy Care; ESEQ = Exercise Self-Efficacy Questionnaire; ET = Exercise Therapy; EurQoL = European Quality of Life Questionnaire; FABQ = Fear Avoidance Beliefs Questionnaire; GDS = Geriatric Depression Scale; GSC = General Strength and Conditioning; HADS = Hospital Anxiety and Depression Scale; HLC = Health Locus of Control questionnaire; IPAC = International Physical Activity Questionnaire—Short Form; LBOSQ = Low Back Outcome Scale Questionnaire; LBRS = Low Back Rating Scale; LL = Lower Limb; MAI = Multidimensional Adherence Index; MCMT = Motor Control Exercise and Manual Therapy; MSI = Movement System Impairment-Based Classification Model; MSPQ = Modified Somatic Perception Questionnaire; NC = Non-Classification-Specific Treatment; nsCLBP = Non-Specific CLBP; N(P)RS = Numeric (Pain Rating Scale); OEES = Outcome Expectations for Exercise Scale; (m)ODI = (modified) Oswestry Disability Index; PALQ = Physical Activity Level Questionnaire; PANAS = Positive and Negative Affect Schedule; PCS = Pain Catastrophising Scale; PDI = Psychological Distress Inventory; PGRS = Pain Graphic Rating Scale; PT = Physiotherapist; PSFS = Patient-Specific Functional Scale; PSQ = Patient Satisfaction Questionnaire; QBPDS = Quebec Back Pain Disability Scale; RMDQ = Roland–Morris Disability Questionnaire; SF-36 = Short-Form Health Survey; SIRAS = Sports Injury Rehabilitation Adherence Scale; SIRBS = Sports Injury Rehabilitation Beliefs Scale; SPPB = Short Physical Performance Battery; TSK = Tampa Scale for Kinesiophobia; TSRQ = Treatment Self-Regulation Questionnaire; VAS = Visual Analog Scale; WAI = Work Alliance Inventory; w/wo = With or Without.

**Table 4:** Results ROB2.

Study Author, Year	Bias Arising from the Randomisation Process	Bias Due to Deviations from Intended Interventions	Bias Due to Missing Data	Bias in Measurement of Outcomes	Bias in Selection of Reported Results	Overall Risk of Bias
Azevedo et al., 2021 [47]	Low	Some concerns	Low	Low	Low	Some concerns
Coppack et al., 2012 [48]	Low	Some concerns	Some concerns	Low	Low	Some concerns
Feng et al., 2025 [56]	Low	Some concerns	Low	Low	Low	Some concerns
Friedrich et al., 1998 [49]	Low	High	Low	Low	Low	High
Lonsdale et al., 2017 [50]	Some concerns	High	Some concerns	Low	Low	High
Mannion et al., 2009 [51]	High	Some concerns	Some concerns	Low	Some concerns	High
Owen et al., 2022 [52]	Low	Some concerns	High	Low	Low	High
Özden et al., 2022 [55]	Low	Some concerns	Low	Low	Low	Some concerns
Van Dillen et al., 2016 [53]	Low	Some concerns	Low	Low	Low	Some concerns

**Table 5:** Results of CASP cohort studies.

Study Author, Year	1	2	3	4	5a	5b	6a	6b	7	8	9	10	11	12	Overall Risk
Dhondt et al., 2020 [54]	Y	Y	? ^13^	Y	Y	Y	Y	Y	Y	Y	Y	Y	Y	Y	Low
Rainville et al., 1997 [57]	Y	Y	? ^14^	Y	Y	Y	? ^15^	Y	Y ^16^	Y	Y	Y	Y	Y ^17^	Low

Y = yes; N = no; ? = unclear. 1. Did the study address a clearly focused issue?; 2. Was the cohort recruited in an acceptable way?; 3. Was the exposure accurately measured to minimise bias?; 4. Was the outcome accurately measured to minimise bias?; 5a. Have the authors identified all important confounding factors?; 5b. Have they taken account of the confounding factors in the design and/or analysis?; 6a. Was the follow up of the subjects complete enough?; 6b. Was the follow up of the subjects long enough?; 7. What are the results of this study?; 8. How precise are the results?; 9. Do you believe the results?; 10. Can the results be applied to the local population?; 11. Do the results of this study fit with other available evidence?; 12. What are the implications of this study for practice?; 13. Same exposure as outcome?; 14. Self-reported questionnaire (financial compensation); 15. Only most patients completed the follow-up period, some were lost to follow-up.; 16. Adherence is associated with rating of change, which is linked to illness perception and outcome expectations.; 17. Common sense model might be helpful to address perceptions and improve adherence.

**Table 6:** Results of CASP qualitative studies.

Study Author, Year	1	2	3	4	5	6	7	8	9	10	Overall Risk
Boutevillain et al., 2017 [39]	Y	Y	Y	Y	Y	?	Y	Y	Y	Y	Low
Gilanyi et al., 2024 [46]	Y	Y	Y	Y	Y	Y	Y	Y	Y	Y	Low
Liddle et al., 2007 [40]	Y	Y	? ^11^	? ^13^	Y	?	?	? ^17^	Y	Y	Moderate
Mathy et al., 2015 [41]	Y	Y	Y	Y	? ^14^	?	Y	? ^18^	Y	Y	Low
Palazzo et al., 2016 [42]	Y	Y	Y	Y	Y	?	Y	Y	Y	Y	Low
Slade, 2009 (listen) [43]	Y	Y	Y	Y	Y	?	N	? ^19^	Y	Y	Low
Slade et al., 2009 (people) [44]	Y	Y	? ^12^	Y	? ^15^	?	Y	? ^20^	? ^21^	Y	Moderate
Stilwell et al., 2017 [45]	Y	Y	Y	Y	? ^16^	?	?	Y	Y	Y	Low

Y = yes; N = no; ? = unclear. 1. Was there a clear statement of the aims of the research? 2. Is a qualitative methodology appropriate? (Is qualitative research the right method for the goal and does the research interpret actions/experiencer of participants?); 3. Was the research design appropriate to address the aims of the research? 4. Was the recruitment strategy appropriate to the aims of the research? 5. Was the data collected in a way that addressed the research issue? 6. Has the relationship between researcher and participants been adequately considered? (bias? how did the researcher respond, were changes in design considered?); 7. Have ethical issues been taken into consideration? 8. Was the data analysis sufficiently rigorous? 9. Is there a clear statement of findings? 10. How valuable is the research? 11. The specific qualitative design is not clearly described within the study; 12. The study does not provide details on the specific research design used; however, interviews are conducted to understand the participants’ perspectives aligns with the stated aims; 13. Details on the recruitment process and sampling strategy are lacking; 14. The study does state that interviews were used to collect data; however, more specifics on the interview guide and data collection are not provided; 15. Specifics on the interview and data collection procedures are not provided rigorously; 16. Semi-structured interview; transcribed but based on researchers’ own notes; 17. The specific analytic approach and process are not described in detail; 18. Not enough specifics are given on the data analysis process; 19. Not sufficiently elaborated on the methods for data analysis; 20. Data analysis processes are not sufficiently detailed; 21. The results do not provide information about the study’s findings or whether they were discussed in relation to the original research question(s).

## Data Availability

This review was prospectively registered in PROSPERO (ID: CRD42024504308).

## Abbreviations

The following abbreviations are used in this manuscript:

ARP	Activity-Related Pain questionnaire
BARSE	Barriers Self-Efficacy Scale
BBQ	Back Belief Questionnaire
BDI	Beck Depression Inventory
BIPQ	Brief Illness Perception Questionnaire
BREQ-2	Behavioural Regulation in Exercise Questionnaire
CG	Control Group
CNS	Central nervous system
CONNECT	Communication Style and Exercise Compliance in Physiotherapy
d	Days
CS	Classification-Specific Treatment
EARS	Exercise Adherence Rating Scale
EBPC	Evidence-Based Physiotherapy Care
ESEQ	Exercise Self-efficacy Questionnaire
ET	Exercise Therapy
EurQoL	European Quality of Life Questionnaire
FABQ	Fear Avoidance Beliefs Questionnaire
FU	Follow-Up
GDS	Geriatric Depression Scale
GSC	General Strength and Conditioning
h	Hours
HADS	Hospital Anxiety and Depression Scale
HEP	Home-Exercise Program
HLC	Health Locus of Control questionnaire
IPAC	International Physical Activity Questionnaire—Short Form
LBOSQ	Low Back Outcome Scale Questionnaire
LBRS	Low Back Rating Scale
LL	Lower Limb
MAI	Multidimensional Adherence Index
MCMT	Motor Control Exercise and Manual Therapy
MSI	Movement System Impairment-Based Classification Model
MSPQ	Modified Somatic Perception Questionnaire
NC	Non-Classification-Specific Treatment
nsCLBP	Non-Specific CLBP
N(P)RS	Numeric (Pain Rating Scale)
OEES	Outcome Expectations for Exercise Scale
(m)ODI	(modified) Oswestry Disability Index
PALQ	Physical Activity Level Questionnaire
PANAS	Positive and Negative Affect Schedule
PCS	Pain Catastrophising Scale
PDI	Psychological Distress Inventory
PGRS	Pain Graphic Rating Scale
PT	Physiotherapist
PSFS	Patient-Specific Functional Scale
PSQ	Patient Satisfaction Questionnaire
QBPDS	Quebec Back Pain Disability Scale
RMDQ	Roland–Morris Disability Questionnaire
SF-36	Short-Form Health Survey
SIRAS	Sports Injury Rehabilitation Adherence Scale
SIRBS	Sports Injury Rehabilitation Beliefs Scale
SPPB	Short Physical Performance Battery
TSK	Tampa Scale for Kinesiophobia
TSRQ	Treatment Self-Regulation Questionnaire
WAI	Work Alliance Inventory
w/wo	With or Without

## Appendix A

Full search strategy on PubMed:

(chronic AND (low OR lower OR spinal OR lumbar OR lumbalgia OR spine OR back) AND (pain OR pain [Mesh] OR low back pain [MeSH Terms] OR back pain [MeSH Terms])) AND (exercise OR training OR exercise therapy [MeSH Terms] OR exercise [MeSH Terms] OR exercise movement techniques [MeSH Terms] OR telerehabilitation OR self-management OR telerehabilitation [MeSH Terms] OR Self-Management [Mesh]) AND (attitude to health [MeSH Terms] OR health belief model [MeSH Terms] OR compliance OR adherence OR compliancy OR contributor* OR barrier* OR facilitator* OR motivation OR knowledge OR attitude* OR belief* OR behavi* OR awareness)

Full search strategy on Web of Science:

ALL = ((chronic AND (low OR lower OR spinal OR lumbar OR lumbalgia OR spine OR back) AND pain) AND (exercise OR training OR “exercise therapy” OR telerehabilitation OR self-management) AND (compliance OR adherence OR compliancy OR contributor* OR barrier* OR facilitator* OR motivation OR knowledge* OR attitude* OR belief* OR behavi* OR awareness))

Full search strategy on SCOPUS:

TITLE-ABS-KEY ((chronic AND (low OR lower OR spinal OR lumbar OR lumbalgia OR spine OR back) AND (pain)) AND (exercise OR training OR telerehabilitation OR self-management) AND (compliance OR adherence OR compliancy OR contributor* OR barrier* OR facilitator* OR motivation OR knowledge OR attitude* OR belief* OR behavi* OR awareness))

## Appendix B

**Table A1:** Intervention-related characteristics.

Reference Author (Country; Year)	Sample (Mean ± SD Age (Years); % ♀; Pain Duration (Months); Pain Intensity (NRS))	Intervention Control Group	Intervention Experimental Group	Duration; Follow-Up
**Quantitative**				
Azevedo (Brazil; 2021) [47]	148 nsCLBP; group 1: 39.6 ± 13.1 group 2: 42.9 ± 12.8; CG: 66.7; EC: 86.9; 3/11	Stretching + strengthening exercises; lumbar + abdominal muscles, lower limbs, HEP	Education, modification of performance of daily activities, specific movement and posture exercises, HEP	12 s, 8 w CG: HEP 1–2 days per week EG: HEP ≥ once a day on days without therapy
Coppack (UK; 2012) [48]	48 CLBP (32.9 ± 7.9); 6% ♀; >31.6; pain intensity not mentioned	CG 1; therapist-led ET CG 2; non-therapist-led ET	Goal setting + ET; individual and group-based submaximal, incremental exercise	3 w, 5 d/w Daily ten 30 min sessions; hydrotherapy, active recovery sessions, relaxation periods Day 6 and 11; FU; addition of new goals
Dhondt (Belgium; 2020) [54]	273 CLBP (40.3 ± 10.7); 58.1% ♀; 3–6 (n = 20), 6–9 (n = 29), 9–12 (n = 35), >12 (n = 159); pain intensity not mentioned	N.A.	Outpatient multimodal program; proprioception, coordination, stability, strength; functional exercises Individual; home-exercise booklet	24 w; 36 sessions (3 evaluation sessions, 33 treatment sessions; one information session, 3 ergonomic sessions, 29 exercise sessions); 2 × 2 h/w
Feng (China; 2025) [56]	78 CLBP; Contr. 27 ± 7; 17% ♀; 24; mild (n = 16), moderate (n = 20), severe (n = 3) Exp: 22 ± 15; 22% ♀; 30; mild (n = 20), moderate (n = 17), severe (n = 2)	Usual care therapy, including patient education and paper handouts describing home exercises	Patient education, health coaching, and structured exercise program delivered through mobile health (mHealth) apps	8 sessions, 1 x/w; assessment at baseline, after 4 weeks (during treatment) and after 8 weeks (post treatment)
Friedrich (Austria; 1998) [49]	93 CLBP; Contr: 44.88 ± 10.96; 44.9% ♀ Exp: 43.27 ± 10.37; 56.8% ♀; CG: 46.1; EG: 50.6; CG 5.5/10, EG 5/10	Standard exercise program; individual, submaximal, gradually increased	Combined exercise (=control) and motivation program; information strategies, reinforcement strategies	10 treatment sessions (25 min, 2–3 x/w), daily home exercises, 4- and 12-m FU
Lonsdale (Ireland; 2017) [50]	255 CLBP (contr: (46.71 ± 13.48); 52% ♀); (exp: (44.11 ± 12.96); 56% ♀) 53 PT (contr: (32.24 ± 5.26); 79% ♀); (exp: (31.92 ± 4.70); 71% ♀); >3; CG 5.8/10, EG 5.5/10	1 h refresher workshop for PT on evidence-based physiotherapy for CLBP	Extra 8 h of communication skills training (CONNECT training)	12-week clinic-based treatment, follow-up 24 weeks since start
Mannion (Switzerland; 2009) [51]	31 nsCLBP (44.0 ±12.3) 65.6% ♀; 92; 3–8/10	N.A.	Spine segmental stabilisation exercises; integration into functional activities; HEP	9 w, 1 x/w HEP: 10 × 10 repetitions, ten times a day (20–25 min/day)
Owen (Australia; 2022) [52]	40 nsCLBP; MCMT: 35 ± 4; 50% ♀ GSC: 35 ± 5; 45% ♀; Undefined duration; CG 4.3/10, EG 5/10	Motor control exercise and manual therapy (MCMT); pain-contingent progression	General strength and conditioning (GSC); aerobic + resistance exercises, time-contingent progression	MCMT: 6 m, 12× 30 min one-on-one physiotherapy sessions GCS: 6 m, 52× 1 h one-on-one supervised gym-based sessions + 20–40 min home-based aerobic exercise 2 x/w
Özden (Turkey, 2022) [55]	50 CLBP; Exp: 40.1 ± 1.6; 56% ♀; 20.6 ± 26.9; 3.8 ± 1.9 Contr: 42.3 ± 1.6; 64% ♀; 22.4 ± 29.8; 3.4 ± 2.8	Video-exercise-based telerehabilitation software called Fizyoweb	Paper-based conventional rehabilitation	8 w, assessment at baseline and after 8 weeks
Rainville (USA; 1997) [57]	192 CLBP; compensation group: 39; 53% ♀; non-comp. group: 43; 58% ♀; 45; 5–6.8/10	N.A.	Group therapy: flexibility, strength, endurance; behavioural techniques; Level 1: 1 h stretching + 1 h strengthening + endurance; Level 2: 45′ stretching, 1 h strengthening, 1 h aerobic training	Level 1: 2 h PT 3× per week; 5–6 sessions Level 2: 2 h + 45 min PT 3× per week; 5 weeks
Van Dillen (USA; 2016) [53]	101 CLBP; Exp: 43.5; 50% ♀ Contr: 42.5; 75% ♀; 96–156; 3/10	Non-classification-specific (NC): education, exercise, performance training; HEP	Classification-specific (CS): education, exercise, performance training HEP; progression based on ability to perform the appropriate number of repetitions	1 h sessions weekly, 6 weeks treatment, follow-up at 6 and 12 months after ending treatment
**Qualitative**				
**Reference Author (Country; Year)**	**Sample (Mean ± SD Age (Years); % ♀; Pain Duration (Months); Intensity (VAS))**	**Intervention-Related Characteristics**		**Outcome Assessment**
Boutevillain (2017) [39]	29 CLBP; (20–30 y n = 3; 31–40 y n = 10; 41–55 y n = 16); 34.5% ♀; >60; <5/10 (31%), >5/10 (69%)	Physical activity in primary and secondary care (not further specified)		Focus groups and individual interviews
Gilanyi (2024) [12]	14 CLBP (20–30 y n = 4, 31–40 y n = 1, 41–50 y n = 1, >51 y n = 5, unknown n = 3); 50% ♀; 0–5 (43%), 6–10 (57%)	Participants had participated in an exercise program (not further specified)		Focus groups and individual interviews
Liddle (2007) [40]	18 nsCLBP; (<20 y n = 1; 20–24 y n = 5; 41–55 y n= 9; 56–65 y n = 3); 77.8% ♀; Undefined duration and intensity	Treatment from a qualified health professional that had included advice and exercise		Focus groups
Mathy (2015) [41]	30 nsCLBP (42 ± 11.5); 53.3% ♀, 40% defined themselves as sporty; 6–420; undefined duration and intensity	Enrolled in a multidisciplinary program, such as the multidisciplinary back school program		Semi-structured interviews
Palazzo (2016) [42]	29 CLBP (20–24 y n = 10; 41–60 y n = 11; 61–85 y n = 8); 58.6% ♀; 13–104; undefined pain intensity	Home-based daily exercises for at least 2 months, learned during supervised sessions in physical therapy department, and received a brochure of prescribed exercises. According to the medical situation and the patient’s socio-professional status, patients followed an out- or inpatient rehabilitation. The programs all included group cognitive behavioural interventions to manage fear-avoidance beliefs, and individual psychological management was proposed if necessary		Semi-structured interviews
Slade (2009) (listen) [43]	18 nsCLBP (51.2 ± 9.5); 66.7% ♀; undefined duration and pain intensity	Participants had participated in an exercise program (not further specified)		Focus groups
Slade (2009) (people) [44]	18 nsCLBP (51.2 ± 9.5); 66.7% ♀; undefined duration and pain intensity	Participants had participated in an exercise program (not further specified)		Focus groups
Stilwell (2017) [45]	6 nsCLBP (34.5 ± 14.4); 50% ♀; 120; undefined pain intensity	Exercise instructions or advice in the past six months (not further specified)		Semi-structured interviews

♀ = females; ARP = Activity-Related Pain questionnaire; BARSE = Barriers Self-Efficacy Scale; BBQ = Back Belief Questionnaire; BDI = Beck Depression Inventory; BIPQ = Brief Illness Perception Questionnaire; BREQ-2 = Behavioural Regulation in Exercise Questionnaire; CNS = Central Nervous System; CONNECT = Communication Style and Exercise Compliance in Physiotherapy; CS = Classification-Specific Treatment; d = Days; EBPC = Evidence-Based Physiotherapy Care; ESEQ = Exercise Self-Efficacy Questionnaire; ET = Exercise Therapy; EurQoL = European Quality of Life Questionnaire; FABQ = Fear Avoidance Beliefs Questionnaire; FU = Follow-Up; GDS = Geriatric Depression Scale; GSC = General Strength and Conditioning; h = Hours; HADS = Hospital Anxiety and Depression Scale; HEP = Home-Exercise Program HLC = Health Locus of Control 1uestionnaire; IPAC = International Physical Activity Questionnaire—Short Form; LBOSQ= Low Back Outcome Scale Questionnaire; LBRS = Low Back Rating Scale; LL = Lower Limb; MAI = Multidimensional Adherence Index; MCMT = Motor Control Exercise and Manual Therapy; MSI = Movement System Impairment-Based Classification Model; MSPQ = Modified Somatic Perception Questionnaire; N.A. = not applicable; NC = Non-Classification-Specific Treatment; nsCLBP = Non-Specific CLBP; N(P)RS = Numeric (Pain Rating Scale); OEES = Outcome Expectations for Exercise Scale; (m)ODI = (modified) Oswestry Disability Index; PALQ = Physical Activity Level Questionnaire; PANAS = Positive and Negative Affect Schedule; PCS = Pain Catastrophising Scale; PDI = Psychological Distress Inventory; PGRS = Pain Graphic Rating Scale; PT = Physiotherapist; PSFS = Patient-Specific Functional Scale; PSQ = Patient Satisfaction Questionnaire; QBPDS = Quebec Back Pain Disability Scale; RMDQ = Roland–Morris Disability Questionnaire; SF-36 = Short-Form Health Survey; SIRAS = Sports Injury Rehabilitation Adherence Scale; SIRBS = Sports Injury Rehabilitation Beliefs Scale; SPPB = Short Physical Performance Battery; TSK = Tampa Scale for Kinesiophobia; TSRQ = Treatment Self-Regulation Questionnaire; WAI = Work Alliance Inventory; w/wo = With or Without.

## Appendix C

**Table A2:** GRADE quantitative studies.

Outcome	Study Design	Risk of Bias	Heterogeneity of Results (Consistency)	Relevance of Evidence (Directness)	Modifying Factors (Precision, Publication Bias)	Confidence Level
Intervention characteristics						
Setting	High certainty; RCT	Serious concerns; high RoB	N.A.	No concerns; evidence is directly relevant to the review question	Moderate concerns; low sample size (n = 93), no CI described, no publication bias	Low
Progression	High certainty; RCT	Some concerns; moderate RoB	N.A.	No concerns; evidence is directly relevant to the review question	Moderate concerns; low sample size (n = 48), wide CI (97.5%), no publication bias	Moderate
Modalities	High certainty; RCT	Some concerns; moderate RoB	N.A.	No concerns; evidence is directly relevant to the review question	Moderate concerns; low sample size (n = 50), no CI described, no publication bias	Moderate
Internal factors						
Beliefs	Moderate; one RCT	Some concerns; high (RCT)	Minor concerns; different results but not contradictory, both no description of CI, but resp. *p* < 0.05 and *p* < 0.01	No concerns; evidence is directly relevant to the review question	Serious concerns; small sample size (n = 40 + 192), no description of CI	Low
Psychosocial	Moderate; one RCT, one cohort	Some concerns; moderate (RCT) and low RoB (cohort)	Minor concerns; different results but not contradictory, both used the same CI (95%)	No concerns; evidence is directly relevant to the review question	Minor concerns; sufficient sample size (n = 148 + 273) but wide CI (95%), no publication bias	Moderate
Symptoms and impairments	Moderate; two RCT’s, one cohort	Some concerns; moderate (RCT) and low RoB (cohort)	Serious concerns; contradictory results (disability as hampering and favouring)	No concerns; evidence is directly relevant to the review question	Minor concerns; sufficient sample size (n = 192 + 101 + 148) but wide CI (95%), no publication bias	Low
Confidence	Moderate; one RCT, two cohorts	Serious concerns; high (RCT), moderate (RCT) and low RoB (cohort)	Minor concerns; similar results, but different CI (95% and 97.5%)	No concerns; evidence is directly relevant to the review question	Minor concerns; sufficient sample size (n = 48 + 273 + 37) but wide CI (85%), no publication bias	Low
Patient-related characteristics	Moderate; two cohorts	Some concerns; moderate (RCT) and low RoB (cohorts)	Serious concerns; contradictory results (increased age as hampering and favouring)	No concerns; evidence is directly relevant to the review question	Minor concerns; sufficient sample size (n = 273 + 192) but wide CI (95%), no publication bias	Low
External factors						
HCP characteristics	High certainty; RCT	Serious concerns; high RoB	N.A.	No concerns; evidence is directly relevant to the review question	Minor concerns; sufficient sample size (n = 255) but wide CI (95%), no publication bias	Moderate

CI = Confidence Interval; HCP = Healthcare Professional; RCT = Randomised Controlled Trial; N.A. = not applicable; RoB = Risk of Bias.

**Table A3:** GRADE-CERQual qualitative studies.

Outcome	Methodological limitations	Coherence	Adequacy of Data	Relevance	Confidence Level
Intervention characteristics					
Modalities	No concerns (all low RoB based on CASP tool)	Minor concerns (studies provide partially different, but complementary results (e.g., multimedia support and telerehabilitation), but they do not undermine each other)	Minor concerns (4 studies, low number of participants (n = 29 + 6 + 29 + 14 resp.), multiple data sources (interviews, focus groups, questionnaires))	No concerns (similar population, setting, and context, and generalisable)	Moderate; outcome is supported by a reasonable number of studies, but limited data slightly reduce confidence
Program design	Minor concerns (one study showed moderate RoB)	Minor concerns (studies provide partially different, but complementary results (e.g., boring and monotonous), but they do not undermine each other)	Minor concerns (5 studies, moderate number of participants (n = 29 + 18 + 6 + 29 + 14 resp.), multiple data sources (interviews, focus groups, questionnaires))	No concerns (similar population, setting, and context, and generalisable)	Moderate; outcome is supported by a reasonable number of studies, but some methodological limitations and limited data slightly reduce confidence
Setting	Minor concerns (one study showed moderate RoB)	Moderate concerns (studies provide similar results, but one contradictory results: group setting and individual preferences are both classified as favouring)	Minor concerns (6 studies, moderate number of participants (n = 29 + 18 + 18 + 6 + 29 + 14 resp.), multiple data sources (interviews, focus groups, questionnaires))	No concerns (similar population, setting, and context, and generalisable)	Moderate; outcome is supported by a reasonable number of studies, but some methodological limitations and limited data slightly reduce confidence
Progression	Minor concerns (one study showed moderate RoB)	No concerns (studies provide the same results)	Minor concerns (4 studies, low number of participants (n = 18 + 6 + 30 + 14), multiple data sources (interviews and focus groups))	No concerns (similar population, setting, and context, and generalisable)	Moderate; outcome is supported by a reasonable number of studies, but some methodological limitations and limited data slightly reduce confidence
HEP	No concerns (all low RoB based on CASP tool)	No concerns (studies provide the same results)	Moderate concerns (2 studies, low number of participants (n = 18 + 29), multiple data sources (interviews and focus groups))	No concerns (similar population, setting, and context, and generalisable)	Moderate; outcome is supported by a reasonable number of studies, but limited data reduces confidence
Follow-up	Minor concerns (one study showed moderate RoB)	No concerns (studies provide the same results)	Minor concerns (5 studies, moderate number of participants (n = 29 + 18 + 6 + 29 resp.), multiple data sources (interviews, focus groups, questionnaires))	No concerns (similar population, setting, and context, and generalisable)	Moderate; outcome is supported by a reasonable number of studies, but some methodological limitations and limited data slightly reduce confidence
Feedback	No concerns (low RoB based on CASP tool)	No concerns (since there are no multiple studies to compare, internal coherence within the study was assessed; data sources align with outcomes and consistent interpretations)	Serious concerns (only one study, low number of participants (n = 29))	No concerns (similar population, setting, and context, and generalisable)	Low; the outcome is well supported with minimal concerns, but only includes a single study
Internal factors					
Beliefs	No concerns (all low RoB based on CASP tool)	Minor concerns (studies provide partially different, but complementary results (e.g., false beliefs exercise importance and passive “fix”), but they do not undermine each other)	Minor concerns (5 studies, moderate number of participants (n = 29 + 30 + 6 + 29 + 14 resp.), multiple data sources (interviews, focus groups, questionnaires))	No concerns (similar population, setting, and context, and generalisable)	Moderate; outcome is supported by a reasonable number of studies, but incoherence and limited data reduce confidence
Psychosocial	Minor concerns (one study showed moderate RoB)	Minor concerns (studies provide partially different, but complementary results (e.g., kinesiophobia and fear avoidance), but they do not undermine each other)	Low concerns (7 studies, reasonable number of participants (n = 29 + 18 + 30 + 18 + 6 + 29 + 14), multiple data sources (interviews, focus groups, questionnaires))	No concerns (similar population, setting, and context, and generalisable)	Moderate; outcome is supported by a reasonable number of studies, but some methodological limitations and incoherence reduce confidence
Symptoms and impairments	Minor concerns (one study showed moderate RoB)	Minor concerns (studies provide partially different, but complementary results (e.g., decreased pain and improved MODI), but they do not undermine each other)	Minor concerns (5 studies, moderate number of participants (n = 29 + 18 + 30 + 29 + 14 resp.), multiple data sources (interviews, focus groups, questionnaires))	No concerns (similar population, setting, and context, and generalisable)	Low; outcome is supported by a reasonable number of studies, but some methodological limitations, incoherence, and limited data reduce confidence
Confidence	Minor concerns (one study showed moderate RoB)	Minor concerns (studies provide partially different, but complementary results (e.g., confidence and ability to perform), but they do not undermine each other)	Minor concerns (4 studies, low number of participants (n = 30 + 18 + 29 + 14 resp.), multiple data sources (interviews, focus groups, questionnaires))	No concerns (similar population, setting, and context, and generalisable)	Low; outcome is supported by a reasonable number of studies, but some methodological limitations, incoherence, and limited data reduce confidence
Patient-related characteristics	No concerns (low RoB based on CASP tool)	Minor concerns (studies provide different results, but they do not complement or undermine each other)	Moderate concerns (2 studies, low number of participants (n = 29 + 14), multiple data sources (interviews and focus groups))	No concerns (similar population, setting, and context, and generalisable)	Moderate; outcome is supported by a reasonable number of studies, but incoherence and limited data reduce confidence
External factors					
HCP characteristics	Minor concerns (two study showed moderate RoB)	Minor concerns (studies provide partially different, but complementary results (e.g., supervision, support and involved HCP), but they do not undermine each other)	Low concerns (8 studies, reasonable number of participants (n = 29 + 18 + 30 + 18 + 18 + 6 + 29 + 14), multiple data sources (interviews, focus groups, questionnaires))	No concerns (similar population, setting, and context, and generalisable)	Moderate; outcome is supported by a reasonable number of studies, but some methodological limitations and incoherence reduce confidence
Environment	Minor concerns (one study showed moderate RoB)	No concerns (studies provide the same results)	Minor concerns (5 studies, moderate number of participants (n = 29 + 30 + 18 + 29 + 14 resp.), multiple data sources (interviews, focus groups, questionnaires))	No concerns (similar population, setting, and context, and generalisable)	Moderate; outcome is supported by a reasonable number of studies, but some methodological limitations and incoherence reduce confidence
Time	Minor concerns (one study showed moderate RoB)	No concerns (studies provide the same results)	Minor concerns (6 studies, moderate number of participants (n = 29 + 30 + 18 +18 + 29 + 14 resp.), multiple data sources (interviews, focus groups, questionnaires))	No concerns (similar population, setting, and context, and generalisable)	Moderate; outcome is supported by a reasonable number of studies, but some methodological limitations and incoherence reduce confidence

CI = Confidence Interval; HCP = Healthcare Professional; HEP = Home-Exercise Program; RCT = Randomised Controlled Trial; RoB = Risk of Bias.
